# An experimental model of rhinovirus induced chronic obstructive pulmonary disease exacerbations: a pilot study

**DOI:** 10.1186/1465-9921-7-116

**Published:** 2006-09-06

**Authors:** Patrick Mallia, Simon D Message, Tatiana Kebadze, Hayley L Parker, Onn M Kon, Sebastian L Johnston

**Affiliations:** 1 Department of Respiratory Medicine, National Heart and Lung Institute and Wright Fleming Institute of Infection & Immunity, Imperial College London, UK; 2 St Mary's NHS Trust, Praed Street, London, UK

## Abstract

**Background:**

Acute exacerbations of COPD are a major cause of morbidity, mortality and hospitalisation. Respiratory viruses are associated with the majority of exacerbations but a causal relationship has not been demonstrated and the mechanisms of virus-induced exacerbations are poorly understood. Development of a human experimental model would provide evidence of causation and would greatly facilitate understanding mechanisms, but no such model exists.

**Methods:**

We aimed to evaluate the feasibility of developing an experimental model of rhinovirus induced COPD exacerbations and to assess safety of rhinovirus infection in COPD patients. We carried out a pilot virus dose escalating study to assess the minimum dose of rhinovirus 16 required to induce experimental rhinovirus infection in subjects with COPD (GOLD stage II). Outcomes were assessed by monitoring of upper and lower respiratory tract symptoms, lung function, and virus replication and inflammatory responses in nasal lavage.

**Results:**

All 4 subjects developed symptomatic colds with the lowest dose of virus tested, associated with evidence of viral replication and increased pro-inflammatory cytokines in nasal lavage. These were accompanied by significant increases in lower respiratory tract symptoms and reductions in PEF and FEV_1_. There were no severe exacerbations or other adverse events.

**Conclusion:**

Low dose experimental rhinovirus infection in patients with COPD induces symptoms and lung function changes typical of an acute exacerbation of COPD, appears safe, and provides preliminary evidence of causation.

## Background

Chronic Obstructive Pulmonary Disease (COPD) is predicted to become the 3^rd ^leading cause of death worldwide by 2020 [1]. Much of the morbidity, mortality and health care costs of COPD are associated with acute exacerbations[2]. Treatments for COPD exacerbations are only partially effective, have significant side effects and do not address specific mechanisms involved in its pathogenesis. Bacterial infections are associated with around 50% of COPD exacerbations and although studies have demonstrated clinical improvement with antibacterial therapy, their therapeutic impact is still disappointing[3,4]. Recent studies report symptoms of virus infection precede two thirds of COPD exacerbations[5] and viruses can be detected in the majority [5-8]. The majority of viruses detected in these studies were rhinoviruses, however a causal relationship between rhinovirus infection and COPD exacerbations has not been established.

Development of new therapies for COPD will require understanding mechanisms of virus-induced lower airway inflammation to identify molecular therapeutic targets. However, little is known about the pathophysiological changes occurring in the lower respiratory tract during COPD exacerbations. In asthma experimental rhinovirus infection has provided valuable insights into the mechanisms linking virus infections to asthma exacerbation [9-14]. The development of an experimental model of rhinovirus-induced COPD exacerbation would be a major step forward in COPD research by providing evidence of a causal relationship between rhinovirus infection and exacerbations, thereby providing impetus to efforts to develop antiviral therapy[15,16]. Such a model would also permit detailed studies of the pathogenesis of COPD exacerbation in a manner not possible with naturally occurring exacerbations. Experimental rhinovirus infection has never previously been carried out in patients with COPD and its effect in this patient group is not known. Safety is the primary concern in all such studies and particularly in relation to COPD as, compared with the mild asthmatics, COPD patients are older, have more severe airway obstruction and less reversibility and are smokers or ex-smokers.

To investigate the feasibility and safety of developing a model of COPD exacerbation and to investigate a causative role of virus infection we conducted a virus dose-escalating pilot study in which we inoculated subjects with COPD with rhinovirus 16 (RV16) and assessed changes in upper and lower respiratory tract symptoms, lung function, and virus replication and inflammatory responses in nasal lavage.

## Methods

### Pilot study design

As there is no precedent for such a study in COPD patients, we designed this pilot study based on published literature and previous experience with challenge studies in normal volunteers and asthmatics. We elected to carry out a virus dose-escalating study to determine the lowest dose of virus that would induce colds in COPD patients. Studies in asthmatic volunteers have inoculated between 100 and 10,000 virus units (tissue culture infective doses 50% [TCID_50_])[9,15]. As this inoculum has not been previously administered to COPD patients and its safety in this patient group was unknown, an initial low dose of 10 TCID_50 _of RV16 was selected as the starting dose. We developed a protocol whereby 5 subjects would be inoculated and if the criteria for completion were not achieved then 100 TCID_50 _would be inoculated into a subsequent group of 5 subjects, followed by 5-fold increasing doses of virus until the criteria for completion of the study were satisfied. The protocol defined criteria for completion of the study were:

1. Colds in ≥ 50% of subjects according to clinical criteria and ≥ 80% evidence of infection according to virological criteria (detecting virus in nasal secretions or a four-fold or greater serum neutralizing antibody response in serum) *and:*

2. An acute exacerbation of COPD (as defined using the lower respiratory tract scoring system) in ≥ 80% of subjects *and:*

3. No severe exacerbations or other adverse events.

#### Study subjects

Subjects were recruited from the Chest Clinic, St Mary's Hospital, London, UK and from local General Practices and fulfilled the inclusion and exclusion criteria in Table 1. Ethical approval was obtained from the Local Research Ethics Committee and informed consent obtained from all subjects.

**Table 1:** Inclusion/exclusion criteria for study subjects.

• Age 40–75 years.
• No history of asthma or allergic rhinitis.
• Not atopic on skin testing.
• Current or ex-smokers with at least 20 pack years cumulative smoking.
• Post-bronchodilator FEV_1 _≤ 80% and ≥ 50% predicted for age and height.
• Post-bronchodilator FEV_1_/FVC ratio less than 70% predicted.
• β-agonist reversibility of less than 12%.
• Absence of a current or previous history of bronchiectasis, carcinoma of the bronchus or other significant respiratory disease (other than COPD).
• Absence of significant systemic disease.
• No COPD exacerbation or respiratory tract infection within the previous 8 weeks.
• Serum antibodies to rhinovirus 16 at screening in a titre <1:2.
• No treatment with oral, inhaled or nasal topical steroids, long-acting β-agonists or tiotropium in the previous 3 months.

### Experimental infection protocol

At an initial screening visit suitability for the study was assessed and symptom diary cards and home spirometry commenced. Inoculation was carried out 2 weeks after screening on study day 0 after spirometry and nasal lavage were performed and blood drawn for baseline serology. The subjects were seen daily for clinical review and nasal lavage on the 8 days post-inoculation and on day 11. Clinic spirometry was performed on study days 4, 7 and 11. At a final visit 6 weeks after inoculation convalescent nasal lavage and serum were collected and spirometry performed.

### Clinical procedures

#### Symptom scores

Subjects completed daily diary cards of upper and lower respiratory tract symptoms from 2 weeks prior to until 6 weeks after the day of inoculation.

#### Upper respiratory tract symptoms

The scoring system and clinical criteria for a cold were derived from Jackson *et al*[17]. The subjects recorded the following symptoms on a scale of 0 (no symptoms) to 3 (severe) – sneezing, runny nose, blocked nose, sore throat or hoarse voice, headache or face pain, generally unwell, fever or shivery, cough. A cold was considered to be present if 2 of the following 3 criteria were present[17]:

1. A cumulative symptom score of at least 14 over a 6-day period.

2. The subjective impression of a cold.

3. Rhinorrhoea present on at least 3 days.

#### Lower respiratory tract symptoms

The most commonly adopted definitions of COPD exacerbations rely on identifying a worsening of the previous stable state based on reporting of increased symptoms[4,18,19]. We used a scoring system (adapted from Calverley *et al*[20]) with which subjects quantified the severity of lower respiratory tract symptoms (Table 2). Based on clinical study scoring systems an exacerbation was defined as an increase over baseline of at least 2 points on 2 consecutive days[4,18-20]. To correct for baseline symptoms, the mean score for each patient on days -6 to 0 was subtracted from post-inoculation scores for both upper and lower respiratory tract scores.

**Table 2:** Lower respiratory tract symptom scoring system.

	SCORE				
SYMPTOMS	0	1	2	3	4
SHORTNESS OF BREATH	Not breathless	On moderate exertion	On mild exertion	On minimal exertion	At rest
WHEEZE	No wheeze	On moderate exertion	On mild exertion	On minimal exertion	At rest
COUGH	No cough	Mild	Moderate	Severe	
SPUTUM QUANTITY (PER 24 HRS)	None	Minimal (<30 ml)	Moderate (30–100 ml)	Large (>100 ml)	
SPUTUM QUALITY	None	Mucoid (clear)	Mucopurulent (yellow)	Purulent (green)	

#### Definition of a severe exacerbation

As the primary aim of the study was to evaluate the safety of experimental rhinovirus infection in COPD subjects, we used the following criteria for defining a severe exacerbation.

1. An increase in shortness of breath score by 2 points or more on 2 consecutive days.

2. An increase in wheeze score by 3 points or more on 2 consecutive days.

3. A fall in FEV_1 _or PEF of 40% or more from baseline.

4. The subject developed a subjective feeling of severe exacerbation and wished to have treatment.

5. The study doctor decided that the subject had a severe exacerbation and required treatment.

If any subject fulfilled any 2 of these criteria he/she would be defined as having a severe exacerbation and would be withdrawn from the study and treatment instituted according to the clinical judgement of the investigators.

#### Virus inoculation

Details regarding the preparation and safety testing of the RV16 inoculum used in this study have been published[21]. The virus was diluted in a total volume of 4 ml of 0.9% saline and inoculated via the nasal route using an atomizer (No. 286; DeVilbiss Co., Heston UK) to spray the virus into each nostril. The subjects were instructed to inhale deeply through the nose simultaneously with activation of the atomizer. This procedure was repeated a number of times until all the inoculum was instilled.

#### Nasal lavage

Nasal lavage was performed by instilling 2.5 mL of 0.9% saline into each nostril, holding for 5 seconds and then expelling into a sterile container. Samples were divided into aliquots and frozen at -80°C until analyzed.

#### Spirometry

Clinic spirometry was performed on a Micromedical MicroLab (MicroMedical, Rochester, UK) spirometer according to BTS/ARTP guidelines[22]. At screening spirometry was performed at baseline and 15 minutes after administration of 200 μg salbutamol via metered dose inhaler and large volume spacer to assess reversibility. Repeat measurements were made on the same spirometer on study days 4, 7, and 11 and at 6 weeks. Subjects carried out daily home spirometry on a portable spirometer (MicroSpirometer; MicroMedical) performing 3 maximal forced expirations at the same time each day and the highest FEV_1_, PEF and FVC were recorded. Baseline lung function was taken as the mean value of the recordings on days -6 to 0.

### Laboratory analysis

#### Virus culture

Nasal lavages were inoculated onto Ohio HeLa cell cultures, and RV infection detected by presence of typical cytopathic effect. Cultures were examined daily for up to 7 days and if no CPE was observed were passaged up to 2 times further. Cultures were regarded as negative if they showed no cytopathic effect after the 2nd further passage. The serotype of the cultured viruses was confirmed by neutralization using RV16 specific antiserum (ATCC V-105-501-558; Bethesda, MD, USA)[23]. Assessment of antibody titre was by microneutralization test using previously published methods[21].

#### RNA extraction and PCR

Viral RNA was extracted from nasal lavage using the QIAmp Viral RNA Mini Kit (Qiagen Ltd) according to the manufacturer's instructions. Samples were analysed for picornaviruses by a reverse transcription method with random hexamers followed by PCR[24]. To differentiate rhinoviruses from other picornaviruses *BglI *enzyme restriction digestion was carried out on the amplicons generated by RT-PCR[25]. Viral load was measured with a real-time quantitative RT-PCR assay[16]. PCR for a panel of other respiratory viruses was carried out as previously described[5], together with PCR for human metapneumovirus adapted from published protocols[26].

#### Detection of cytokines

Cytokines were measured in nasal lavage using the human cytokine 10-plex assay™ (BioSource International). Assays were performed for IL-1, IL-2, IL-4, IL-5, IL-6, IL-8, IL-10, GM-CSF, TNF-α and IFN-γ. Fresh 0.1% dithiothreitol was added to samples at 1:5, samples were vortexed, left for 10 minutes on ice, aliquotted and stored at -80°C. Assays were carried according to the manufacturer's instructions and analysed on the Luminex™ 100 system.

### Statistical analysis

Data are presented as mean (SEM) values. Variables were compared with repeated measures ANOVA or Student's t-test. Differences were considered significant for all statistical tests at p values of less than .05. Analysis was performed using GraphPad Prism version 4.00 for Windows, GraphPad Software, San Diego California USA.

## Results

The first 4 subjects who were recruited and inoculated with 10 TCID_50 _of the virus inoculum achieved all 3 of the study endpoints, and therefore the study was completed at this dose of virus. The clinical characteristics of these subjects are shown in Table 3.

### Induction of symptoms

#### Upper respiratory tract

**Figure 1:**
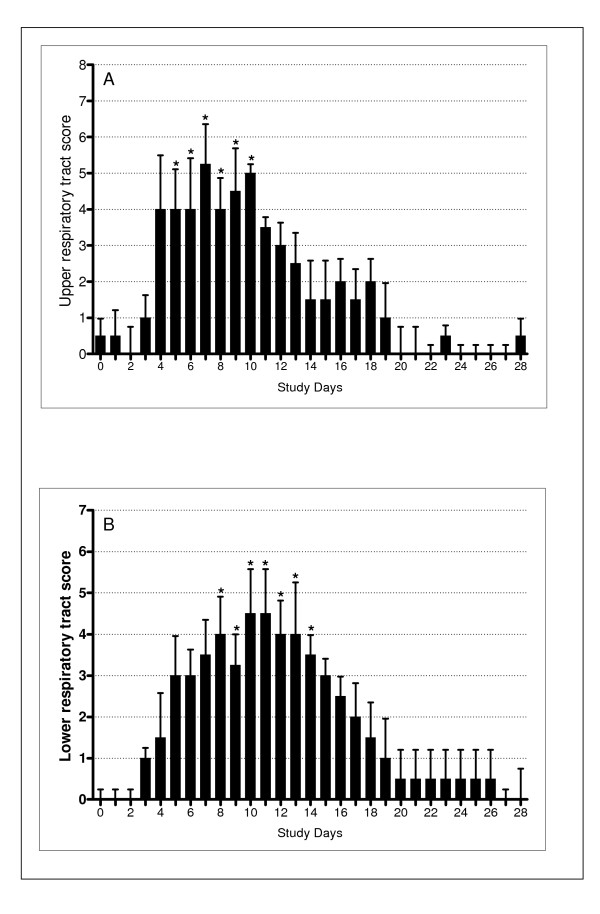
Daily upper and lower respiratory tract scores. (A) Mean total upper respiratory tract symptom scores. Symptoms were significantly increased on days 5 – 10. * indicates p < 0.05 compared to baseline. (B) Lower respiratory tract symptom scores. Symptoms were significantly increased on days 7 – 14. * indicates p < 0.05 compared to baseline. Mean scores on days -6 to 0 were subtracted from post-inoculation scores for both upper and lower respiratory tract scores.

A clinical cold developed in all 4 subjects according to the predetermined clinical criteria; the time course of the upper respiratory tract symptoms is shown in Figure 1A. Upper respiratory tract symptom scores were significantly increased compared to baseline (ANOVA p < 0.0001) on study days 5 to 10 with the peak of cold symptoms occurring on day 7.

#### Lower respiratory tract

**Figure 2:**
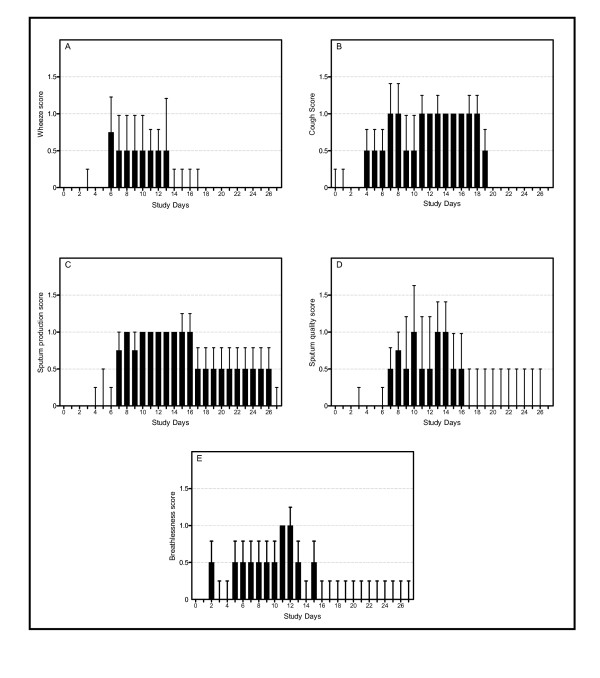
Individual lower respiratory tract symptoms. (A) Wheeze (p = 0.01). (B) Cough (p < 0.001). (C) Sputum production (p = 0.01). (D) Sputum quality (p = 0.19). (E) Shortness of breath (p = 0.82).

All subjects developed an increase in lower respiratory tract symptoms that fulfilled the pre-determined criteria for an exacerbation; the time course for lower respiratory tract scores is shown in Figure 1B. Lower respiratory tract symptom scores were significantly increased compared to baseline (ANOVA p < 0.0001) on days 7 to 14, with peak lower respiratory tract symptoms on days 10 and 11. When the individual symptoms were analysed separately, all five lower respiratory symptom domains increased from baseline (Figure 2), however the increases were only statistically significant for wheeze (ANOVA p = 0.01), cough (ANOVA p < 0.001) and sputum production (ANOVA p = 0.01). In terms of recovery, all symptom domains other than sputum production had recovered to baseline by day 20, however full recovery of sputum production took almost 4 weeks.

### Lung function

**Figure 3:**
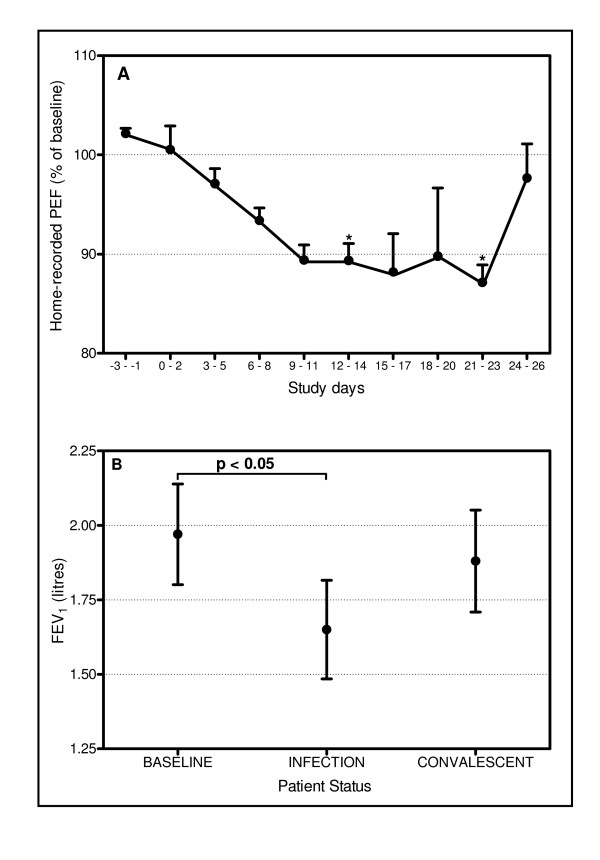
Daily and clinic spirometry measurements. (A) 3 day average of home-recorded PEF. There were significant falls in PEF on days 12 – 14 and 21 – 23. * indicates p < 0.05 compared to baseline. (B) FEV_1 _measured in clinic. There was a significant fall in FEV_1 _compared to baseline (p < 0.05).

Figure 3A shows the home PEF readings expressed as a 3-day average. Home PEF fell progressively from baseline after infection, reaching a nadir of approximately 12% reduction from baseline on days 9–11 through 21–23, before recovering on days 24–26 (ANOVA p = 0.017). PEF was significantly reduced on days 9–11 (p = 0.022) and 21–23 (p = 0.016). There was no statistically significant fall in FEV_1 _measured on the home spirometers. The change in FEV_1 _measured in clinic is shown in Figure 3B. FEV_1 _fell by ~16% from a mean of 1.97L at baseline to 1.65L at infection (p = 0.046).

### Cytokine measurements

**Figure 4:**
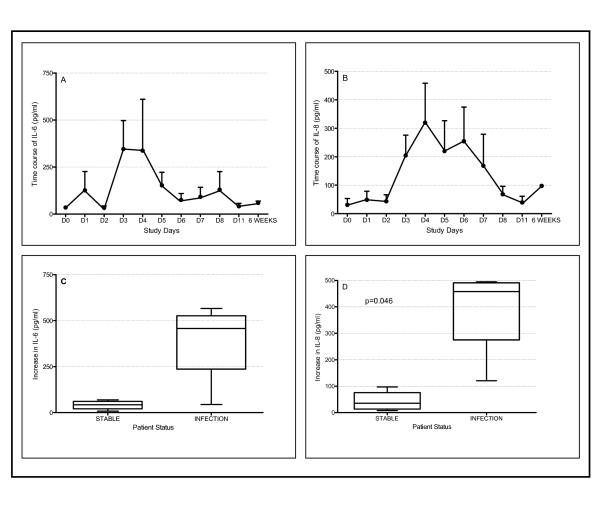
Levels of IL-6 and IL-8 in nasal lavage. (A) Time course of IL-6. (B) Time course of IL-8. (C) Mean levels of IL-6 in nasal lavage when stable and at exacerbation. There was an increase in IL-6 at exacerbation but this was not significant (p = 0.054). (D) Mean levels of IL-8 when stable and at exacerbation. There was a significant increase in IL-8 at exacerbation (p = 0.046).

Among the panel of cytokines measured in nasal lavage fluid changes were only detected in the levels of IL-6 and IL-8 (Figure 4). Figures 4A and 4B show the time course of the changes in IL-6 and IL-8 levels respectively. The peak levels of cytokines after inoculation were compared to baseline. Mean IL-6 levels rose from 40.56 pg/ml to 381.2 pg/ml but this did not reach statistical significance (p = 0.054) (Figure 4C). There was a significant increase in IL-8 from 44.1 pg/ml when stable to 382.8 pg/ml during infection (p = 0.046) (Figure 4D).

### Virology

**Figure 5:**
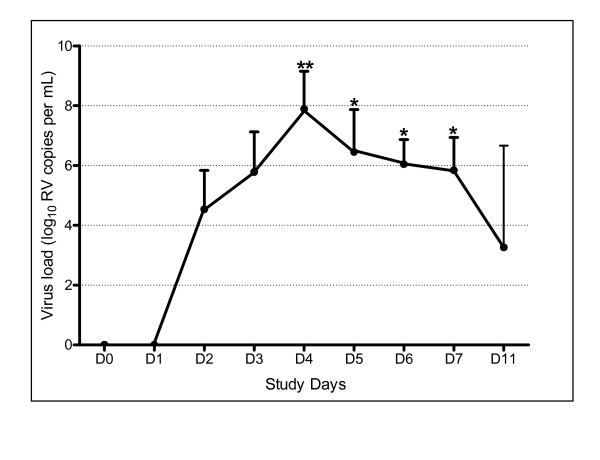
Viral load measured with measured with a real-time quantitative RT-PCR assay. Viral load was significantly increased above baseline on days 4 – 7. ** indicates p < 0.01. * indicates p < 0.05.

All subjects had a negative PCR for picornavirus and the panel of other respiratory viruses on nasal lavage taken on day 0 prior to inoculation. PCR for picornavirus became positive on day 2 in all subjects and remained positive at day 8 (n = 4) and day 11 (n = 2). *BglI *digestion confirmed in all cases that the picornavirus detected by PCR was a rhinovirus. Co-infection was excluded by negative PCR for the other respiratory viruses in the panel. Rhinovirus was cultured from nasal lavage in all subjects and the serotype confirmed to be RV16 by neutralizations with specific RV16 antiserum. All subjects developed positive RV16-specific serologic responses to infection. PCR on convalescent samples taken at 6 weeks was negative for picornavirus. The results of the picornavirus quantitative PCR are shown in Figure 5. There was an increase in viral load (ANOVA p < 0.0001) that was significant on days 4 (p < 0.01) and 5, 6 and 7 (p < 0.05).

#### Safety

No subject fulfilled the criteria for a severe exacerbation or experienced any other adverse events.

## Discussion

We report the first study to experimentally infect COPD patients with a respiratory virus. Our aim was to assess the feasibility and safety of developing a human experimental model of virus-induced COPD exacerbation, and to provide preliminary evidence for a causative role for virus infection in exacerbations. We demonstrated that experimental rhinovirus inoculation of subjects with underlying COPD results in clinical colds, together with increased lower respiratory tract symptoms and falls in PEF.

These data provide preliminary evidence of safety for this experimental model and suggest that it is feasible to further develop the model in larger numbers of subjects. They also provide preliminary evidence for a causal role for virus infections in inducing exacerbations of COPD.

In order to develop new therapies for COPD exacerbations a detailed understanding of the causes of exacerbations, as well as the pathogenic mechanisms is needed. Since studying naturally occurring exacerbations is extremely difficult, development of an experimental model in which causation could be confirmed and in which detailed clinical studies on mechanisms of disease could be carried out, would be a major step forward. Experimental virus infection has been used extensively in both healthy volunteers and asthmatics to study the pathogenesis of the common cold and virus-induced asthma exacerbations, as well as to identify and evaluate potential new treatments for these conditions[11,13,15,27]. Respiratory virus infections are associated with between 40% and 65% of COPD exacerbations [5-8], and rhinoviruses are the most common virus type detected. However the safety of experimental rhinovirus infection in COPD patients has never been evaluated.

Using a low dose of RV16 inoculum we successfully induced colds in COPD patients. In addition to the upper respiratory tract symptoms RV16 infection resulted in a sustained increase in lower respiratory tract symptoms and significant falls in lung function, typical of those seen in naturally occurring exacerbations. Consistent with previous studies of naturally occurring exacerbations increases in symptoms and reduction in lung function persisted for between 2 and 4 weeks from virus inoculation[28].

The changes in PEF (~12%) seen in this study were of similar magnitude to that reported elsewhere[29]. The colds were accompanied by evidence of viral replication and increased pro-inflammatory cytokines in the upper respiratory tract, although the increase in IL-6 levels just failed to reach significance. As the aim of this study was primarily to ascertain the feasibility and safety of RV16 inoculation in COPD we did not carry out lower airway sampling but the results from this study suggest that further studies to evaluate the effect of rhinovirus infection on lower airway inflammation are warranted. Although epidemiological studies have shown an association between virus infection and COPD exacerbation they do not prove causation. This study provides further supportive evidence in addition to epidemiological studies that respiratory virus infection can cause COPD exacerbations. Given that there is data suggesting virus-induced exacerbations are more severe[5], development of effective antiviral strategies is an urgent priority.

The timing of upper respiratory tract and lower respiratory tract symptoms observed in this study may have important implications for diagnostic epidemiology of virus induced COPD exacerbations[5,6]. The virus load in nasal lavage was greatest on day 4, whereas the peak lower respiratory tract symptoms occurred on days 10/11. Assessing the relationship between virus infection and COPD exacerbations depends on sampling for viruses when patients report lower respiratory tract symptoms. Sampling the upper respiratory tract for viruses when patients present with lower respiratory tract symptoms may give a falsely low detection rate, as sampling will likely occur well after the peak of virus load has passed in the upper respiratory tract.

This interpretation is supported by our data as only 2/4 (50%) of the subjects had positive nasal lavage PCR for rhinoviruses on day 11. Further evidence to support this comes from the East London COPD study in which 64% of exacerbations were preceded by colds but a virus was detected in only 39%[5], so the true association of respiratory virus infection and COPD exacerbations is likely even higher than reported. This may also have important implications for therapy of virus-induced COPD exacerbations. The cold symptoms peaked on day 7, but were clearly elevated as early as day 4, co-incident with the peak in virus load, whereas lower respiratory symptoms peaked on days 10 & 11, suggesting that if an effective antiviral or anti-inflammatory agent were administered at the onset of cold symptoms, there is a window of several days for treatment to exert a beneficial effect. There is already data suggesting that early treatment of exacerbations leads to better outcomes[30] and this data should encourage efforts to develop new treatments for exacerbations of COPD.

An unexpected finding of this study was that all subjects developed colds and exacerbations with 10- to 1,000-fold lower doses of virus than used in previous studies in asthmatic and normal volunteers[9,15]. This could suggest that COPD patients have increased susceptibility to virus infection, as has recently been demonstrated in asthma [31,32]. Further studies will be needed to investigate this interesting and potentially very important possibility.

We acknowledge that the conclusions of this study are derived from results on only 4 subjects, however as the pre-determined criteria for termination of the study were reached (infection and exacerbation in 80% of 5 subjects), we were obliged to terminate the study. We chose this study design as previous dose-finding studies in experimental virus infections have used similar patient numbers[33,34]. The data from this study suggests that experimental rhinovirus infection can be used to develop a valid and safe model of COPD exacerbation. Such a model could overcome the many obstacles that investigators face in studying naturally occurring exacerbations including under-reporting of exacerbations[5], delay in presentation[30], varying aetiology, difficulties in sampling the lower airway and variation in timing from onset of exacerbation to clinical assessment and sampling. These difficulties can all be overcome in the experimental setting, leading to high quality, well controlled data that is likely to take us significant steps forward in the search for novel therapies.

## Conclusion

We have shown that experimental rhinovirus infection in COPD patients results in colds that are accompanied by lower respiratory tract symptoms and lung function changes typical of naturally occurring exacerbations. These were associated with evidence of viral replication and inflammatory cytokines in the upper airway. These findings suggest experimental rhinovirus infection has potential as a model of COPD exacerbation.

## Competing interests

SLJ has received research funding from GlaxoSmithKline, Merck & Sanofi-Aventis. SLJ has also received consulting fees from GlaxoSmithKline and fees for speaking from GlaxoSmithKline, Merck, Pfizer & Sanofi-Aventis.

None of the other authors has any competing interests.

## Authors' contributions

PM assisted in the study design, recruited the subjects and carried out the clinical and laboratory procedures.

SM assisted in study design and in clinical and laboratory procedures in the study.

TK carried out the PCRs for respiratory viruses.

HP carried out the human cytokine 10-plex assay™.

OMK was responsible for clinical monitoring of the subjects during the study.

SLJ conceived the study idea and developed the study protocol.

All authors have read and approved the final manuscript.
